# Bioinformatics for Whole-Genome Shotgun Sequencing of Microbial Communities

**DOI:** 10.1371/journal.pcbi.0010024

**Published:** 2005-07-12

**Authors:** Kevin Chen, Lior Pachter

## Abstract

The application of whole-genome shotgun sequencing to microbial communities represents a major development in metagenomics, the study of uncultured microbes via the tools of modern genomic analysis. In the past year, whole-genome shotgun sequencing projects of prokaryotic communities from an acid mine biofilm, the Sargasso Sea, Minnesota farm soil, three deep-sea whale falls, and deep-sea sediments have been reported, adding to previously published work on viral communities from marine and fecal samples. The interpretation of this new kind of data poses a wide variety of exciting and difficult bioinformatics problems. The aim of this review is to introduce the bioinformatics community to this emerging field by surveying existing techniques and promising new approaches for several of the most interesting of these computational problems.

## Introduction

Metagenomics is the application of modern genomics techniques to the study of communities of microbial organisms directly in their natural environments, bypassing the need for isolation and lab cultivation of individual species [1–6]. The field has its roots in the culture-independent retrieval of *16S* rRNA genes, pioneered by Pace and colleagues two decades ago [7]. Since then, metagenomics has revolutionized microbiology by shifting focus away from clonal isolates towards the estimated 99% of microbial species that cannot currently be cultivated [8,9].

A typical metagenomics project begins with the construction of a clone library from DNA sequence retrieved from an environmental sample. Clones are then selected for sequencing using either functional or sequence-based screens. In the functional approach, genes retrieved from the environment are heterologously expressed in a host, such as *Escherichia coli,* and sophisticated functional screens employed to detect clones expressing functions of interest [10–12]. This approach has produced many exciting discoveries and spawned several companies aiming to retrieve marketable natural products from the environment (e.g., Diversa [http://www.diversa.com] and Cubist Pharmaceuticals [http://www.cubist.com]). In the sequence-based approach, clones are selected for sequencing based on the presence of either phylogenetically informative genes, such as *16S,* or other genes of biological interest [13–17]. The most prominent discovery from this approach thus far is the discovery of the proteorhodopsin gene from a marine community [14].

Recently, facilitated by the increasing capacity of sequencing centers, whole-genome shotgun (WGS) sequencing of the entire clone library has emerged as a third approach to metagenomics. Unlike previous approaches, which typically study a single gene or individual genomes, this approach offers a more global view of the community, allowing us to better assess levels of phylogenetic diversity and intraspecies polymorphism, study the full gene complement and metabolic pathways in the community, and in some cases, reconstruct near-complete genome sequences. WGS also has the potential to discover new genes that are too diverged from currently known genes to be amplified with PCR, or heterologously expressed in common hosts, and is especially important in the case of viral communities because of the lack of a universal gene analogous to *16S*.

Nine shotgun sequencing projects of various communities have been completed to date (Table 1). The biological insights from these studies have been well-reviewed elsewhere [3,6]. Here, we highlight just two studies that exemplify the exciting possibilities of the approach. The acid mine biofilm community [18] is an extremely simple model system, consisting of only four dominant species, so a relatively miniscule amount of shotgun sequencing (75 Mbp) was enough to produce two near-complete genome sequences and detailed information about metabolic pathways and strain-level polymorphism. At the other end of the spectrum, the Sargasso Sea community is extremely complex, containing more than 1,800 species [19,20]. Nonetheless, with an enormous amount of sequencing (1.6 Gbp), vast amounts of previously unknown diversity were discovered, including over 1.2 million new genes, 148 new species, and numerous new rhodopsin genes. These results were especially surprising given how well the community had been studied previously, and suggest that equally large amounts of biological diversity await future discovery.

**Table 1:**
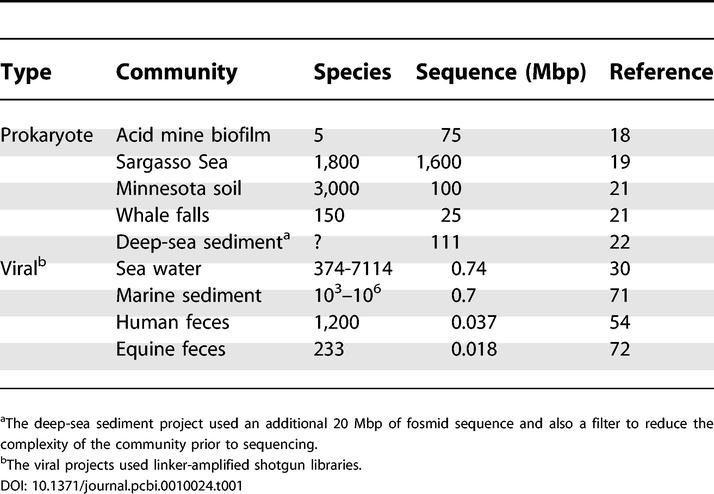
Published Microbial Community Shotgun Sequencing Projects

^a^The deep-sea sediment project used an additional 20 Mbp of fosmid sequence and also a filter to reduce the complexity of the community prior to sequencing.

^b^The viral projects used linker-amplified shotgun libraries.

In this review, we survey several of the most interesting computational problems that arise from WGS sequencing of communities. Traditional approaches to classic bioinformatics problems such as assembly, gene finding, and phylogeny need to be reconsidered in light of this new kind of data, while new problems need to be addressed, including how to compare communities, how to separate sequence from different organisms in silico, and how to model population structures using WGS assembly statistics. We discuss all these problems and their connections to other areas of bioinformatics, such as the assembly of highly polymorphic genomes, gene expression analysis, and supertree methods for phylogenetic reconstruction.

Although we have chosen to focus on the shotgun sequencing approach, we stress that this is only one piece of the exciting field of metagenomics, and that the integration of other techniques such as large-insert clone sequencing, microarray analysis, and proteomics will be vital to achieve a comprehensive view of microbial communities.

## Assembling Communities

The retrieval of nearly complete genomes from the environment without prior lab cultivation is one of the most spectacular results of metagenomics to date. A fundamental limit on the WGS approach is that we can only expect to assemble genomes that constitute a significant fraction of the community [21]. Filtration and normalization techniques that enrich the library for certain low-abundance species, a common technique in the sequencing of symbionts, are thus of vital importance when genome assembly is a primary goal [22,23].

When a closely related, fully sequenced genome is available, comparative assembly can easily be performed by extracting the homologous sequence and assembling it with either a comparative assembler [24] or an alignment program that can handle draft sequence [25,26]. This approach is standard and has been used many times for mixed sequence from multiple species ([19,27]; E. Allen, unpublished data).

In the absence of an appropriate template genome, traditional overlap–layout–consensus assembly [28] can be done, augmented by an additional binning step, in which scaffolds (contiguous sequence with gaps of approximately known size) are separated into species-specific “bins.” The first issue that needs to be overcome is the increased amount of polymorphism, since each read will typically be sampled from a different individual in the population. Second, highly conserved sequence shared between different species can seed contigs and cause false overlaps. In some communities, even phylogenetically distant genomes can share a large number of genes [29]. Careful study of the optimal overlap parameters for separating out sequences at different phylogenetic distances is important, and has been carried out for viral communities [30], but not yet for prokaryotes.

The assembly of communities has strong similarities to the assembly of highly polymorphic diploid eukaryotes, such as *Ciona savigny* [26] and *Candida albicans* [31], if we view prokaryotic strains as analogous to eukaryotic haplotypes. The main difference is that in a microbial community, the number of strains is unknown and potentially large, and their relative abundance is also unknown and potentially skewed, while in most eukaryotes we know a priori the number of haplotypes and their relative abundance. This disadvantage is mitigated somewhat by the small size and relative lack of repetitive sequence in prokaryotic and viral genomes, so that the issue of distinguishing alleles from paralogs and polymorphism from repetitive sequence is less acute.

Thus far, both community assembly and polymorphic eukaryotic assembly have been performed by running a single-genome assembler, such as the Celera assembler [32] or Jazz [33], and then manually post-processing the resulting scaffolds to correct assembly errors. Contigs erroneously split apart because of polymorphism are reconnected, and contigs based on false overlaps are broken apart. Not surprisingly, ad hoc heuristics must be employed to adapt programs optimized for single-genome assembly: the Celera assembler, for instance, treats high-depth contigs associated with abundant species as repetitive sequence.

A promising direction for both these problems is co-assembly, in which two very closely related genomes (or even two assemblies of the same genome) are assembled concurrently, using alignment information to complement mate-pair information in ordering scaffolds and correcting assembly errors in a structured, automated way. Thus far, the only published work on this problem is that of Sundararajan et al. [26], and even then, only for two genomes. For three or more genomes, even the multiple alignment problem for draft sequence is not solved. Large-insert clone sequence will also be very useful since the entire clone comes from a single strain or haplotype [22,34].

After scaffolds have been constructed, the next step is to bin the scaffolds according to species or phylogenetic clade. The gold standard for binning is the presence of a phylogenetically informative gene. *16S* rRNA, though universal, is decidedly not single copy, so it is important to also consider other genes, such as *RecA, EFG, EFTu,* and *HSP70* [19]. In the absence of one of these genes, genome signatures such as dinucleotide frequencies, codon bias, and GC-content, developed by Karlin and others in a long series of papers [35–38], can be used. These signatures appear to work for scaffolds on the order of 50 kbp in length, and, importantly, they seem to correlate only with phylogenetic relatedness and not with the environment [36]. There is a web server, Tetra, that computes tetranucleotide frequencies for metagenomics projects [39,40].

An additional source of evidence unique to WGS data is scaffold read depth, which is expected to be proportional to species abundance and thus can be used to separate high-abundance from low-abundance species. Subtleties can arise, however, since a variable polymorphism rate across a genome can cause conserved regions to be covered at high depth and variable regions to be covered at low depth.

For some applications, completely accurate binning may not be required. For example, gene finders based on hidden Markov models (HMMs) require training data from closely related species. The accuracy of the gene finder might be improved by additional training data, even if it is not from exactly the same species. One could even imagine running the following iterative algorithm: find a set of putative genes, construct gene trees with them, use the trees to crudely bin the scaffolds, retrain the gene finder, and repeat.

To conclude our discussion of assembly, we consider the important question of determining how much to sequence in order to assemble genomes. When sequencing a single genome, the Lander–Waterman model based on the assumptions of independent and random reads implies that the coverage of each base is distributed according to a Poisson distribution with parameter *c* (the coverage). Defining *n_k_* to be the number of bases covered exactly *k* times and *G* to be the genome size, we have


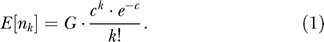


First consider the problem of assembling the most abundant genome at, say, 8× coverage. In the worst case, all species are present in equal abundance. The Lander–Waterman equation holds with *G* replaced by the sum of the sizes of all genomes of species in the community (sometimes called the metagenome). For the soil community, we have *n*_2_ = 300,000 and G = 10^8^/*c,* so the equation implies a coverage of 0.006 and a total of 133 Gbp of sequence needed to assemble the most abundant genome at 8× coverage, disregarding the problem of binning. The total metagenome size predicted is *G* = 16.7 Gbp, corresponding to 2,800 *E. coli–*sized genomes, which is consistent with previous estimates of soil microbial diversity and the *16S* survey.

For the lower bound, we make the additional assumptions that all genomes have length 6 Mbp and that a single dominant species contributes all the overlaps in the assembly. The same equation implies that 2 Gbp of additional sequence is required for assembly at 8× coverage. This number is about twice that calculated from the *16S* survey, but this might be explained by preferential amplification bias in PCR.

We performed similar calculations for the three whale fall communities. In addition, we considered the problem of assembling all genomes in these communities. Since the *16S* survey indicated that three dominant species constitute approximately half the total abundance and all other species have roughly equal abundance, the Lander–Waterman model implies that the expected coverage should be distributed as the mixture of two Poissons with equal weight. The results of these calculations are summarized in Table 2. Similar results were obtained by Venter et al. [19] and Breitbart et al. [30], and there is also software for performing such calculations (http://phage.sdsu.edu/phaccs) [41].

**Table 2:**
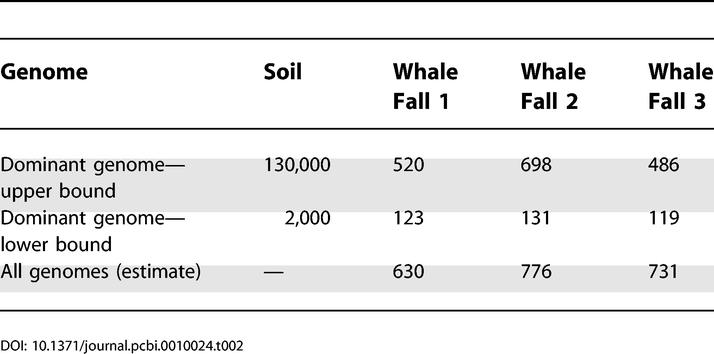
Bounds on Amount of Sequence Needed to Assemble Genomes (in Mbp)

## Comparative Metagenomics

Gene finding is a fundamental goal in virtually all metagenomics projects, regardless of whether complete genome sequences can be assembled or not. If large scaffolds can be retrieved and binned, excellent HMM-based microbial gene finders such as FGENESB (http://www.softberry.com) and GLIMMER [42,43] can be used, in combination with expectation-maximization (EM) techniques for unsupervised training of the HMM parameters [44,45]. At the other extreme, we have unassembled reads of roughly 700 bp. These make up 50% of the total reads in the Sargasso Sea dataset and 100% in soil. Since prokaryotic genes are typically short, lack introns, and occur at high density (roughly one in 1,000 bp), each read is likely to contain a significant portion of a gene. For these reads, HMM techniques are unlikely to be successful, leaving BLAST search against a protein database or the community itself as the only realistic alternative.

There have been two simulation studies verifying the accuracy of BLAST for gene finding with single reads [21,46], though it is difficult to make this kind of experiment convincing, since the accuracy of the method is almost entirely dependent on the availability of closely related sequences in the database. We are not aware of any studies on the accuracy of HMM-based techniques on sequences significantly shorter than a whole genome, so we undertook a simple experiment ourselves. We sampled simulated “contigs” of length 10 kb from the complete genome sequence of *Thermoplasma volcanium* [47]. For each, we predicted genes using GLIMMER trained only on long open reading frames in the contig, and compared these to the GLIMMER predictions when trained on long open reading frames from the entire genome. We found that the results were surprisingly good. Of 92 genes completely contained in the ten simulated contigs, 86 were predicted exactly correctly. There were 16 genes that crossed the boundaries of the contigs, and GLIMMER was able to find truncated genes for seven of these. On the other hand, five of the completely spurious predictions all came from the same contig, which suggests that HMM accuracy may not be uniform over the length of the genome. More detailed studies on this problem are needed to relate the length of assembled contigs to the accuracy of the gene finder. An interesting direction is to attempt to recover more partial genes that overlap contig boundaries, firstly, by making the gene finder aware that genes on the boundary may be truncated and, secondly, by taking advantage of base quality scores for lower quality sequence at the ends of contigs. Another interesting research problem is to fine-tune gene finders for viral genomes.

The gene complement of a microbial community can be used as a fingerprint of a community, allowing us to compare different communities in a gene-centric, as opposed to genome-centric, fashion [21]. In this method, predicted genes are blasted against the COGs [48] or KEGG [49,50] databases and each community is assigned a fingerprint vector with entries corresponding to the number of hits to each COGs or KEGG category. It is also possible to cluster the COGs hits by function in order to compare the communities at a higher level.

**Figure 1:**
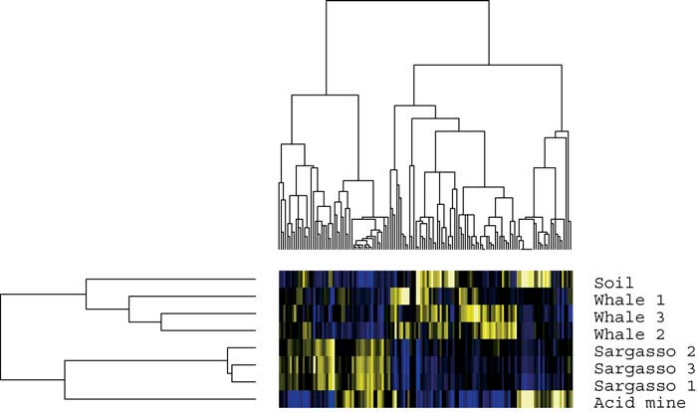
Blue-Yellow Microarray Figure Applied to KEGG Vectors for Four Metagenomics Projects The whale-fall and Sargasso sea data are partitioned into three different samples each. The rows correspond to the different datasets and the columns to the 137 KEGG categories. Blue corresponds to underrepresentation and yellow to overrepresentation. Note that some branch lengths have been adjusted for visualization purposes and do not correspond to an actual meaningful distance.

Fingerprint vectors are analogous to gene-expression-level vectors in microarray analysis and any of the standard gene expression clustering methods can be used [51]. We first replicated the result of [21] by directly applying popular the off-the-shelf gene expression tools, CLUSTER and TreeView [52], to perform single-linkage hierarchical clustering on the KEGG vectors from several communities (Figure 1).

**Figure 2:**
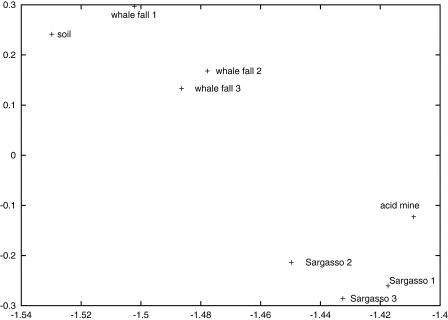
Projection of the KEGG Vectors on the First Two Principle Components

Although the neat tree structure of the blue-yellow microarray figure (Figure 1) looks appealing, it can also be misleading at times because of the properties of UPGMA (unweighted pair group method with arithmetic mean) clustering. To check this, we applied principle components analysis to the fingerprint vectors (Figure 2). While the high-level result is similar, the principle components analysis shows that the clustering of the communities is somewhat more ambiguous than Figure 1 might suggest. For instance, note the surprising proximity of whale-fall sample 1 to the soil sample.

In addition to clustering, principle components analysis has the additional advantage that dimensions of the principle components with high magnitude may correspond to COGs or KEGG sequences of interest, and the principle components themselves may correspond to interesting pathways or functions. This has not yet been fully explored and could potentially be a source of new functional pathways in communities.

Finally, since fingerprinting has been advocated as an alternative to genome assembly when the amount of sequence required for assembly is very high [21], an important issue that needs to be discussed is how much sequence is required to fingerprint. In the same spirit as our Lander–Waterman calculations (equation 1), we estimate this quantity using the observation that the number of genes per shotgun read is very close to one [21,46]. Assuming a uniform species abundance distribution, we get the classic coupon collector's problem [53], in which the number of reads needed to collect a fraction *f* of the *N* genes in the community is exactly


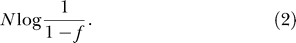


Applying equation 2 to the soil community, if we assume 4,000 genes per genome and 3,000 genomes, then sampling half the genes would require 6 Gbp of sequencing, comparable to the lower bound on the amount of sequence needed to assemble the dominant genome (Table 2).

Based on these observations, it seems that it may be too early to conclude that fingerprinting is a powerful way of comparing communities. We also note that fingerprinting is difficult for viruses, since 65% of predicted genes from the viral community sequencing projects have no homolog in the databases [6]. However, similar techniques have been used to compare the species, as opposed to their gene complements, across different viral communities [54].

## Phylogeny and Community Diversity

If complete gene sequences can be recovered from the community, classic multiple sequence alignment (MSA) [55] and phylogeny algorithms [56] can be applied. If only partial genes are available, phylogenetic reconstruction is still reasonably straightforward if there is already a database of nearly complete sequences, as with *16S* [57] or *RecA* (http://www.tigr.org/_jeisen/RecA/RecA.html). The partial sequences can then be aligned against the complete ones, and the phylogenetic assignment performed by finding the closest sequences in the database [58]. Even for such genes, however, it is plausible to imagine a future in which the majority of genes in the database are in fact partial environmental sequences—at one point, for instance, the Sargasso Sea dataset made up 5% of the total genes in GenBank and a large number of these were unassembled reads. Alternatively, metagenomics projects may discover a highly diverged group of species that may not align well to existing sequences. In these scenarios, it will be necessary to have good MSA and phylogeny tools for partial sequences, even for these “universal” genes.

The case of viral phylogeny is more complex, firstly, because it is not clear that all viruses are related by a tree, and, secondly, because viral taxonomy has traditionally not been based on molecular sequence data, though the Phage Proteomic Tree [59] represents a step in the direction of sequence-based taxonomy. Viral taxonomy is at a very early stage of development, and there is no doubt that culture-independent methods will play an important role in the growth of the field.

Partial sequences are the crux of the phylogeny problem in the context of metagenomics. We are particularly interested in methods for such sequences because they will also be applicable for low-coverage sequencing projects of vertebrates and other species [46,60]. We are not aware of any MSA tools and phylogeny programs that are able to cope with short partial gene fragments, any two of which may fail to have significant overlap. At the alignment stage, we require a semi-global multiple alignment (i.e., terminal gaps are not penalized). The most widely used alignment tools are based on global or local alignments and do not correctly handle partial sequences (an exception is MAP [61]). Since most MSA tools are based on progressive alignment according to a guide tree, it is also important to construct this tree based on pairwise semi-global alignments and conserved terminal *k*-mers, as opposed to the pairwise global or local alignments currently used.

We studied 40 *phosphoglycerate kinase* genes from the soil study and aligned them with MUSCLE [62]. Though not optimized for partial sequences, MUSCLE did a reasonable job, as ascertained by several criteria: the number of internal gaps was small, sequences shorter than the read length had either no beginning gaps or no ending gaps (since the gene length is greater than the read length), and the total length was comparable to related proteins.

Of the 780 pairs of sequences, 95 pairs had overlap of less than 50 amino acids, and of these, 48 pairs had no overlap at all. Thus, we have an extreme instance of the missing data problem, which has been extensively discussed in the phylogenetics literature [63,64]. However, this literature has mostly studied consensus tree methods, and the effect of adding incomplete taxa and/or characters on the accuracy of traditional methods, like maximum likelihood. Relatively little effort has gone into actually finding better methods for tree reconstruction with this kind of data. Supertree methods [65], which attempt to construct trees from multiple subtrees, present one such alternative. One reason these methods have not been widely used in the past in the context of molecular data is the relative lack of maturity of the field as compared with parsimony or likelihood methods. However, encouraging new algorithmic results and software in this area [66–68] should spur renewed work on these types of methods. Supertree methods have also been criticized because incomplete data matrices (e.g., from fossil data) usually do not fit a random and independent missing data model. On the other hand, shotgun sequencing does fit this model and thus would seem an ideal setting for supertree methods. While the data might be too limited to provide completely resolved phylogenies, as previous discussed in the context of binning, even crude trees may be sufficient for certain applications, such as training HMMs.

Finally, with regards to community diversity, one of the advantages of the WGS approach is that it is less biased then PCR, which is known to suffer from a host of problems [69]. Community modeling based on analysis of assembly data within the Lander–Waterman model is beginning to show that species abundance curves are not lognormal as previously thought [41,70], so new methods that take into account these naturally occurring distributions are needed.

## Conclusion

The number of new community shotgun sequencing projects continues to grow, promising to provide vast quantities of sequence data for analysis. Samples are being drawn from macroscopic environments such as the sea and air, as well as from more contained communities such as the human mouth (Table 3). Exciting advances in our understanding of ecosystems, environments, and communities will require creative solutions to numerous new bioinformatics problems. We have briefly mentioned some of these: assembly (can co-assembly techniques be used to assemble polymorphic genomes and complex communities?), binning (what is the best way to combine diverse sources of information to bin scaffolds?), gene finding (how should gene finding programs, which were designed for complete genes and genomes, be adapted for low-coverage sequence?), fingerprinting (which clustering techniques are best suited for discovering novel pathways and functional groups that allow communities to adapt to their environments?), and MSA and phylogeny (how can we best construct trees and alignments from fragmented data?).

**Table 3:**
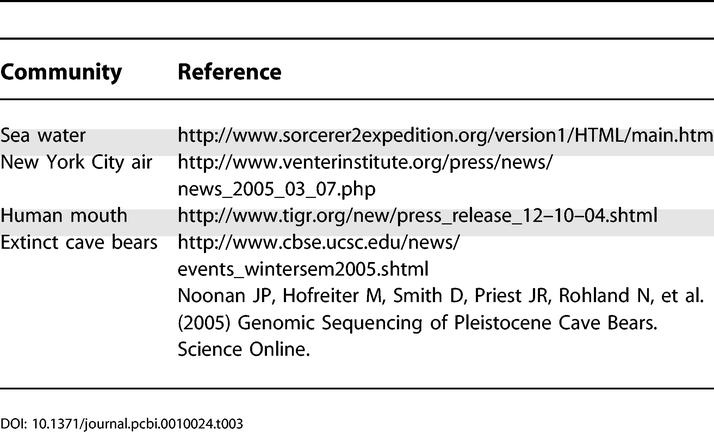
Examples of Ongoing Community WGS Sequencing Projects

Countless more challenges will likely emerge as WGS sequencing approaches are used to tackle increasingly complex communities. The reward for computational biologists who work on these problems will be the satisfaction of contributing to the grand enterprise of understanding the total diversity of life on our planet. 

## Abbreviations

HMM: hidden Markov model
MSA: multiple sequence alignment
WGS: whole-genome shotgun
